# Recirculation: A New Concept to Drive Innovation in Sustainable Product Design for Bio-Based Products

**DOI:** 10.3390/molecules22010048

**Published:** 2016-12-29

**Authors:** James Sherwood, James H. Clark, Thomas J. Farmer, Lorenzo Herrero-Davila, Laurianne Moity

**Affiliations:** Green Chemistry Centre of Excellence, Department of Chemistry, University of York, York YO10 5DD, UK; james.clark@york.ac.uk (J.H.C.); thomas.farmer@york.ac.uk (T.J.F.); lorenzo.herrero-davila@york.ac.uk (L.H.-D.); laurianne.moity@york.ac.uk (L.M.)

**Keywords:** bio-based products, bio-based economy, circular economy, end-of-life, waste, sustainability

## Abstract

Bio-based products are made from renewable materials, offering a promising basis for the production of sustainable chemicals, materials, and more complex articles. However, biomass is not a limitless resource or one without environmental and social impacts. Therefore, while it is important to use biomass and grow a bio-based economy, displacing the unsustainable petroleum basis of energy and chemical production, any resource must be used effectively to reduce waste. Standards have been developed to support the bio-based product market in order to achieve this aim. However, the design of bio-based products has not received the same level of attention. Reported here are the first steps towards the development of a framework of understanding which connects product design to resource efficiency. Research and development scientists and engineers are encouraged to think beyond simple functionality and associate value to the potential of materials in their primary use and beyond.

## 1. Introduction

Faced with the challenges of modern day societal, economic, and ecological needs, there is an obvious need to manage resources more efficiently while ensuring the stability of economic growth. Realizing this, the European Commission issued a strategy for sustainable growth, and how it can be realized with a bio-based economy [1]. There are far-reaching benefits to a bio-based economy, achieved with products made from renewable materials. Resource efficiency, reduced dependency on unsustainable fossil fuels, job opportunities, and climate change mitigation are thought to be attainable, and the impact potentially more beneficial than that of the biofuel sector [2]. The European bio-based product market is projected to grow at 5% per annum, but specific markets, plastics especially, have a growth potential of 16% between 2008 and 2020 for example [3]. Along with bio-based plastics, key areas for growth have been highlighted as bio-based lubricants, bio-based solvents, and bio-based surfactants.

In order to encourage and support this growth, research projects and a standardization program have been used to develop and improve the understanding of bio-based product functionality, the calculation of bio-based content, the understanding of life cycle impacts, and the communication and reporting of these properties. One area that is lacking is guidance for product design, especially as the European bio-based economy is to be immersed into plans for a circular economy. A circular economy uses resource efficiency strategies to minimize waste [4]. How products fulfil their function, meaning both their design and the business model employed to allow the product to deliver its intended service, must be re-evaluated to realize the promise of a circular economy. The choice of materials and how they are joined together has a great effect on the ability to reuse or recycle a product. For the sake of resource efficiency, it is important to extend the lifespan of materials but also maintain the value of those materials after subsequent reuse or recycling.

Bio-based products are made of renewable resources, but this does not mean they can be disposed of under the assumption they biodegrade and cause no harm to the environment. Bio-based products are not necessarily biodegradable, and can be ecotoxic just as petrochemicals can. Furthermore a significant amount of energy and resources are required to cultivate and process biomass, and so to not use that material for as long as possible and in as many uses as possible is a waste of potential, and ultimately, literally a waste.

The European Commission decided that new standards would be beneficial to accelerate the uptake of bio-based products in Europe, thus grow the bio-based economy, and in doing so displace unsustainable fossil derived products [5]. Amongst other things, standards give requirements and test methods to define and measure the properties and characteristics of substances, materials, and products. They are not legally binding in themselves but may support legislation [6]. With respect to bio-based products, the objective of each standard is to improve confidence in new products, and hence encourage trade. In addition, standards are able to harmonize labelling and enable procurement policies to embrace bio-based content and other attributes unique to bio-based products [7].

The European Committee for Standardization (CEN) is responsible for delivering standards describing bio-based products within the European Union. One example of a European standard is EN 16785-1:2015. It is titled “Bio-based products: Bio-based content—Part 1: Determination of the bio-based content using the radiocarbon analysis and elemental analysis”. It is a systematic method of assigning a value of total bio-based content to a bio-based product. EN 16785-1:2015 is an example of a verifiable test method that substantiates claims of bio-based content to the benefit of the market. More European standards relevant to bio-based products are listed in Table 1. Most have been created by the CEN technical committee for bio-based products (CEN/TC 411). Standards are also in development to formally describe the attributes of the four key bio-based product types listed earlier (plastics, lubricants, solvents, and surfactants).

The scientific support for the activities of CEN with regard to bio-based products comes in the form of the Open-Bio (opening bio-based markets via standards, labelling, and procurement) project [9], and the now completed KBBPPS (Knowledge Based Bio-based Products’ Pre-Standardization) project [10]. The consortium for both projects has been led by the Dutch standardization institute (NEN) [11]. Whereas KBBPPS was initiated to conduct pre-standardization research to establish the basis for various test methods, the Open-Bio project operated alongside the standardization process at CEN. Open-Bio has several focus areas, including the determination of bio-based content, functionality testing, end-of-life options (recycling and biodegradation), opportunities for eco-labelling, procurement, and stakeholder acceptance of bio-based products.

## 2. Bio-Based Product Characteristics

### 2.1. Bio-Based Content

The fundamental attribute of a bio-based product is the proportion of renewable material actually contained within it. It is not necessarily true that a bio-based product is completely made of biomass or substances exclusively derived from biomass. In fact the (European) specifications for some bio-based products only require 25% bio-based content. This is true of bio-based solvents (CEN/TS 16766:2015) and bio-based lubricants (EN 16807:2016) in particular. In other instances, a minimum requirement is not even in place, meaning even lower proportions of bio-based content are acceptable. However a minimum bio-based content must be guaranteed, which in business communications can be presented according to a reporting template found in standard EN 16848:2016. In the USA, another system is in operation with a more tailored approach that applies different minimum bio-based content thresholds, each specific to one of many different product types. The result is the BioPreferred catalogue, the purpose of which is to assist a program of mandatory purchasing for bio-based products by US government agencies [12].

**Figure 1:**
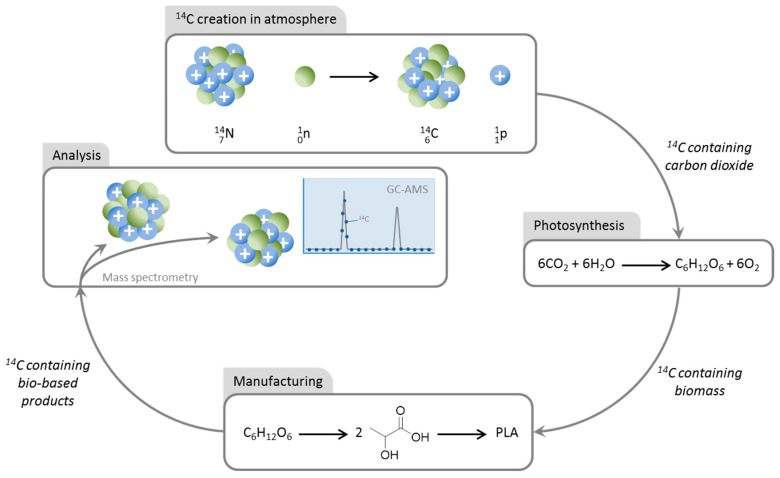
Aspects of the ^14^C radioisotope relevant to bio-based content analysis.

The principle of measuring bio-based content on the basis of carbon atoms is well established. It is the basis of the BioPreferred program. Bio-based carbon content (as it is formally known) is determined using radiocarbon analysis. More famously known in the context of radiocarbon dating, when a bio-based product is analyzed, the ratio of ^14^C to ^12^C isotopes is indicative of the amount of biomass (renewable carbon) compared to fossil carbon (non-renewable carbon). The carbon in biomass crops was recently atmospheric carbon dioxide, and as such is mixed with nitrogen that has been exposed to cosmic ray energy. Nitrogen-14 is converted to ^14^C under these conditions in parts per trillion quantities, oxidized, and incorporated within the bulk atmospheric carbon dioxide (Figure 1). If the ^14^C present in a sample of a bio-based product matches a corrected reference value, it is considered to have 100% bio-based carbon content. If it is a quarter of the reference ^14^C:^12^C ratio, the product is 25% bio-based (on a carbon mass basis), and so on. Methods and their accuracy have been covered by Hooijmans and Norton [13,14,15]. One form of analysis is accelerator mass spectrometry (AMS), where the ^14^C isotope is detected directly in the carbon created when the sample is combusted and the resulting CO_2_ reduced is a series of pre-treatments [16]. Otherwise, ^14^C can be indirectly determined through its radioactive decay. The half-life of ^14^C is 5730 years [17]. Technical specification CEN/TS 16640:2014 documents the proper use of these techniques and how to calculate bio-based carbon content. The standard in use in the USA is ASTM D6866 [18], and is slightly different to the European CEN/TS 16640:2014 in terms of how inorganic carbon is treated.

Total bio-based content (reported on the basis of all atoms) cannot be obtained purely through analysis. The convenience of the ^14^C marker is not repeated in other elements. Stable isotopes vary too much to be of broad use for bio-based products generally, although for specific products they can provide a helpful indication of the origin of a product and sometimes reveal how it was processed [19].

**Figure 2:**
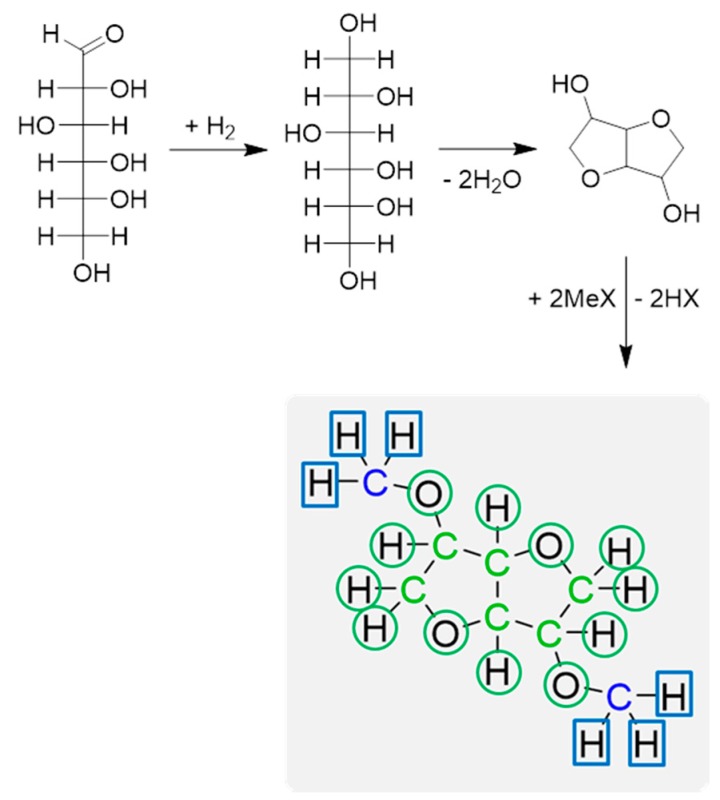
A scheme of a dimethyl isosorbide synthesis, producing a product with 75% bio-based carbon content and 83% total bio-based content. Green atoms are known to be bio-based from direct analysis; blue atoms are known to be not bio-based from analysis; atoms in green circles are assigned to be bio-based; atoms in blue circles are assigned not to be bio-based.

EN 16785-1:2015 presents a method by which the radiocarbon confirmed bio-based carbon content is supplemented by elemental analysis for a declaration of the total bio-based content. In the case of dimethyl isosorbide presented in Figure 2, where isosorbide is methylated, the total bio-based content is 83%. The hydrogen reacted with glucose to produce the intermediate sorbitol is counted as bio-based because the assignment is governed by atom connectivity. The rule of atom connectivity, taken from an Association Chimie Du Végétal (ACDV) principle [20], states atoms covalently bonded to bio-based carbon atoms are also considered to be bio-based. This is a convention that is adopted to align the results of this calculation method to analytical bio-based carbon content data. Strictly speaking, it may not always be correct but other variations of the atom connectivity rules are more difficult to reproduce [21]. The standardized method of calculating total bio-based content is therefore as follows:

- Bio-based carbon content obtained by ^14^C analysis;
- The correct bio-based carbon atoms (shown in green in Figure 2) are assigned from a knowledge of the feedstocks;
- Composition validated with elemental analysis;
- All covalently bonded heteroatoms and hydrogen atoms adopt the origin of the neighboring carbon atom;
- Atoms bonded to a bio-based carbon and a fossil carbon are assigned their origin based on producer knowledge of feedstocks and the synthesis of the product;
- Total bio-based content is calculated on a mass basis.

According to EN 16785-1:2015, the accuracy to which the total bio-based content can be reported depends on the closeness of the match between analytical and theoretical values.

### 2.2. Sustainability and Life Cycle Impacts

A bio-based product is not automatically sustainable, or even more sustainable than a petrochemical product. There is a burden of proof that extends far beyond bio-based content. To begin with the sustainability of the biomass, feedstocks must be questioned. This is a pressing concern given the significant use of biomass for fuels (notably bio-ethanol) but increasingly now also bio-based products. Infamously bioenergy crops have displaced food crops [22], and monoculture farming and deforestation (for palm oil production for example) raises concerns over global biodiversity [23]. Certification exists to verify the sustainability of biomass production [24], as required by legislation such as the European Renewable Energy Directive [25], which is backed by standards [26]. When standards support legislation they are called ‘harmonized standards’. The series of European standards for bio-based products (Table 1) are ‘voluntary standards’. Sustainability criteria for biomass feedstocks and the production of bio-based products have been published as EN 16751:2016. Standard practice for conducting a life cycle assessment (LCA) of bio-based products is also now available (EN 16760:2015).

The use phase of a product can be very specific according to the application and the user. General requirements for bio-based solvents (CEN/TS 16766:2015) list physical properties which can only inform the purchasing decision of a client. Otherwise, the producer can demonstrate the product is ‘fit for purpose’ (where standards are available) but the operator or consumer bears the responsibility to use to product appropriately. It can be especially challenging therefore to demonstrate a bio-based product is used sustainably. Equally, some bio-based products offer enhanced functionality that is not always formally recognized, whether that is through performance criteria in standards or procurement procedures [27].

In contrast to the use phase, end-of-life decisions can be guided more definitively with standards. Here, bio-based products are not a special case in terms of their performance. Biodegradability is dependent on the chemical structure of the material or substance, not its origin. Recycling is limited by material attributes such as thermal stability and the mechanical properties of the recyclate and its complexity rather than the bio-based content. It is true that some bio-based products are not exact copies of large volume conventional products, polylactic acid (PLA) being one example, and so these new types of products may have unforeseen consequences on waste management practices. To build confidence in a bio-based economy, the impact of an increased market presence of bio-based products should be understood. The allowable ‘contamination’ of PET recyclates with PLA without reducing the material performance is a topic of continued interest as PLA gains popularity [28], as is the marine biodegradability of bio-based products [29], a timely investigation given recent reports highlighting the problems of microplastics in the oceans [30].

## 3. Recirculation

### 3.1. Concept

**Figure 3:**
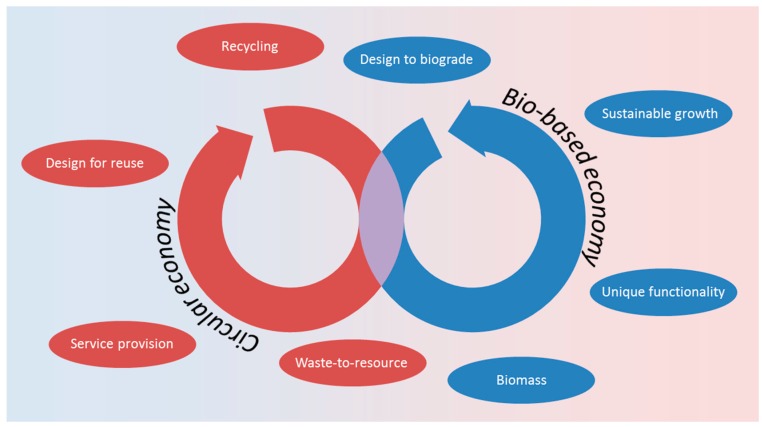
A representation of how the themes of a circular economy relate to those of a bio-based economy [32].

The vocabulary of bio-based products has been defined in EN 16575:2014. This terminology must be revisited to build a basis for product design criteria in a bio-based economy, and also complement the aspirations of a circular economy. A circular economy has been proposed by the European Commission as a means of achieving a resource efficient European economic area [31]. The basic premise of a circular economy is that materials are made from renewable and secondary materials without unnecessary waste. Instead of waste, new feedstocks are generated at the end of a product’s useful lifetime from materials that might have traditionally been sent to landfill. Legislative proposals are focused on reducing waste and pollution, as well as their complementarity with the resource focused initiatives of the bio-based economy (Figure 3).

One key aspect of a circular economy is that it is not achieved simply by increasing recycling rates. It is a misconception to think that simply by increasing the capacity of recycling plants, coupled with more effective recovery of recyclable materials, complete recycling of waste streams will be accomplished. Even for seemingly simple examples of plastic packaging, recycling (especially of domestic waste) is marred by difficulties with separation and (for example, food) contamination. In these instances the sorting and cleaning effort is not always compensated by the reduction in primary product manufacture that recycling provides. Cost benefit analyses show optimum plastic packaging recycling rates are limited to, at best, little more than 50% [33]. This means half of plastic packaging is either (i) designed in such a way that makes recycling difficult; (ii) ends up in complex mixed waste streams from which it cannot be recovered; (iii) is contaminated beyond recovery; or (iv) is made of low market volume materials meaning the recyclate market is also small, with insufficient economic benefit to encourage recycling. Prior to 2014, discussions on the economic benefits to recycling were stronger in what was a high price crude oil market [34]. Now, writing in 2016, at a time of relatively cheap crude oil and natural gas [35,36], the flexible plastic materials that end up in mixed wastes are considered to be unattractive recyclates because of the aforementioned collection and separation issues [34,37].

Europe only recycles 35% of its plastic packaging [38,39]. In other less economically developed regions like India and South Africa the market for used plastic is bigger. The UK for example is mostly exporting waste to Asia for recycling [40]. Whereas bottles and commercial films are examples of easily recycled single layer packaging, many small items of domestic packaging such as multilayer food packaging are not recycled effectively. The priority of food quality, minimizing the spoiling of food, is the most important characteristic of the packaging. Life cycle assessments factoring in the risk of food waste show the use of multilayer non-recyclable packaging has a lower environmental impact overall than less effective single layer packaging [41,42].

Other times, the design of packaging prioritizes labelling and branding above ease of recycling, as is the case for full wrap shrink labels. These full bottle labels are made of a different material to the bio-based polyethylene terephthalate (PET) bottle underneath, often polystyrene or polyvinyl chloride. This is also true of smaller polypropylene adhesive sealed labels but these float on water and are washed into a separate polypropylene recycling stream during waste management [43]. Most present day full wrap shrink labels are not so easily separated from PET and interfere with subsequent color sorting or near infra-red sorting [44]. This example shows that even products made of separate recyclable materials can be inadvertently or carelessly designed to reduce the final recyclability of the article in practice.

Another area of confusion surrounding plastics relates to compostable plastic food packaging, where the end-of-life option is designed to prevent litter and pollution, but consumer misunderstandings can result in non-biodegradable plastic contaminating compost [45], or post-consumer sorting practices excluding all plastic from organic recycling because of the uncertainty over what products are appropriate [46]. Nevertheless, this is only a problem of communication (labeling), and recycling best practice. As renewable and biodegradable plastics such as PLA grow in popularity [28], waste management will treat them more seriously as a valuable source of secondary materials rather than sporadic contamination.

Although the example of plastic packaging has been used here to introduce the topic, it is problematic because of the vast volumes of waste material it generates, the same issue of product design principles overlooking end-of-life options applies to significantly more technical and more valuable products as well. Electrical and electronic equipment (EEE) is made difficult to recycle because of its complexity and how the product is assembled. To be able to easily reclaim the precious metals in waste, EEE would be most helpful in contributing to a circular economy, for many elements have supply risks from the European perspective [47]. The issue of waste EEE recycling [4,48], and elemental sustainability [49], is covered in detail elsewhere. With European Directive 2012/19/EU requiring a 45% rate of waste EEE collection [50], and only two-thirds of collected waste EEE in the European Union actually being recycled (2013 data) [51], there is still much to improve on.

What is important, regardless of the nature of the article, or whether is it bio-based or not, is that in a circular economy the lifespan of materials is maximized by keeping them in use as long as possible (through recycling for instance). A greater association by consumers with the service of products, not the identity of the product itself, must occur to back the consensus that if waste is reduced, and materials are used for longer, then a greater service is obtained from the finite amount of material resource available to us. The design of products to be easily separated and dismantled into their components, each made of the most appropriate material for optimal (planned) end-of-life processing is the basis of what allows us to improve reuse and recycling rates [52], and correct our historically poor use of resources. Changing consumer expectations within a circular economy by offering service-based business models, in effect renting the function of a product [53], might be an even more ambitious exercise.

### 3.2. Definitions

The 12 principles of green chemistry (Table 2) provide a framework for chemical products that are ‘benign by design’ [54]. Low toxicity and biodegradability (where appropriate) reduce the impact of chemistry. The use of renewable feedstocks is also encouraged. The prevention of waste (the first principle of green chemistry) is usually thought of as the materials that are not incorporated into products. For bio-based products, there is an opportunity to define criteria for the optimum use of feedstocks, to create products that do not become waste, but instead have been designed to allow the material they are composed of to remain useful beyond the lifespan of that product. A ‘recirculated’ bio-based product means the material contained within the article is returned back to a usable state, without unnecessarily becoming waste. This helps to maximize material efficiency and reduces pollution and waste (e.g., litter, landfill, net increases in long cycle CO_2_ emissions).

The key to establishing the concept of ‘recirculation’ for bio-based products starts with their design. A formal definition of recirculation was developed in the Open-Bio project to focus efforts towards improving product design so that the materials they are made from do not become redundant (i.e., waste or pollution) after use. The full description is freely available [55]. To understand recirculation, first the nature of feedstocks must be appreciated. ‘Renewable resources’ and ‘renewable feedstocks’ are familiar terms applied to the products of biomass. Their definitions speak of continually replenishing resources or materials. The difference is the former relies on natural processes to regenerate the resource, while the latter specifies that the resource (and source of renewable materials) is biomass.

- Renewable resource [56]: A resource with the ability to be continually replenished by natural processes.
- Renewable material (EN 16575:2014): Material that is composed of biomass and that can be continually replenished.
- Biomass (EN 16575:2014): Material of biological origin excluding material embedded in geological formations and/or fossilized.

Ultimately ‘renewable’ is describing biomass produced with energy captured in photosynthesis. The term ‘resource’ can generally be substituted with ‘feedstock’ and thus is applicable as a description of the input to bio-based product manufacturing. The definitions of ‘renewable resources’ and ‘renewable materials’ could be considered as interchangeable, although the latter might be applied to intermediates or processed biomass. Now having established the context in which the word ‘renewable’ can be used, we see a definition for the term ‘renewable chemical’ also relies on it. The use of ‘renewable’ remains anchored in the resource (biomass) and then transferred to the downstream product. No mention of the sustainability of the biomass is made in these definitions.

- Renewable chemical [57]: A monomer, polymer, plastic, formulated product, or chemical substance produced from renewable biomass.

A bio-based product is produced from renewable material(s) but that does not guarantee that the article contributes to the replenishment of the resources it relies on. In fact, its design may hinder it, but the end-of-life of a bio-based product is not part of its definition.

- Bio-based product (EN 16575:2014): A product wholly or partly derived from biomass.

Here we endorse ‘recirculation’ as an over-arching term to describe bio-based products with end-of-life options that facilitate the continued availability of the material the product is made from (Table 3) [58]. Additionally, a sub-set of definitions have been created to emphasize end-of-life and resource efficiency considerations. Note that these definitions only apply to products, not resources, feedstocks, or materials etc. The wording of the definitions arose from discussions and consultations organized by the Open-Bio project [58].

**Figure 4:**
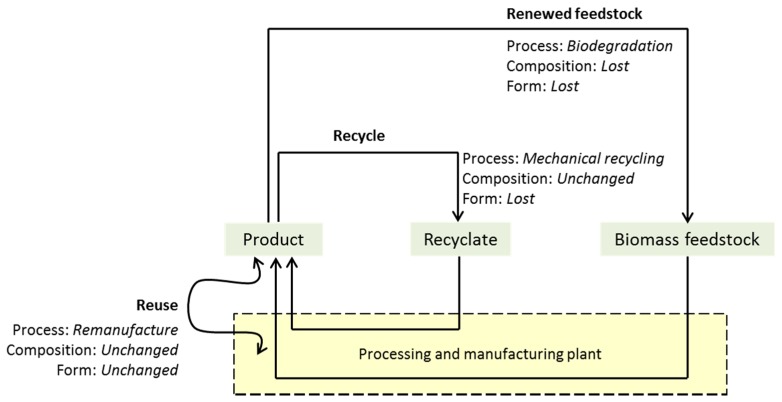
Product recirculation through general examples of reuse, recycle, or feedstock renewal.

How the definitions are applied is governed by the preservation of an article’s form and function. When a product (or a component part) is reused, it retains its form, and in turn its ability to function is maintained. Of course, this requires its chemical composition to have basically remained unchanged. Mechanical recycling removes the form (and hence some value) of the product; melting and extruding or other processes create a batch of recycled material [59], but normally leaves the chemical composition intact. Chemical recycling, the deconstruction of a material into the original intermediates [60], will retain some functionality but polymerization to reform the original polymer is then needed to return the material to use as an end-product. This is most promising for polyesters where mechanical recycling does not produce a high-specification recyclate, as is the case for PLA [61]. Renewability of an article (not a resource or material in this instance) is demonstrated in biological processes where the form and the chemical composition must necessarily be lost. Biodegradation creates and releases carbon dioxide, but only to the effect of completing the carbon cycle for bio-based products. From the definitions in Table 3 a clear hierarchy presents itself, ordered according to how much of the value of a bio-based product is retained after end-of-life waste processing is complete (Figure 4). Reuse is thus preferable to recycling, while the renewal of biomass feedstocks by organic recycling is the least preferred mode of recirculation. Do not mistake this conclusion as suggesting all products should be directly reusable. The application of the product will determine the best end-of-life option. For instance, ultimate biodegradability in the environment has no material value, but is desirable for products used in that context, for instance some types of fishing equipment, the coatings on boat hulls, mulch films, and chainsaw lubricants for the forestry industry that cannot be recovered. Reuse and recycling are intrinsically not viable or prohibitively expensive in these scenarios and so biodegradation is the most favorable end-of-life option.

Some concessions have been made regarding the definitions to accommodate existing vocabulary, in particular those that address the concept of renewability. This definition of ‘renewable’ in the context of material recirculation (Table 3) is specific to 100% bio-based products. This leaves biodegradable fossil derived articles unclassified with respect to any of the three subset definitions of recirculation: namely ‘reusable’, ‘recyclable’, and ‘renewable’. Anaerobic digestion and gasification can be considered as options for the renewal of chemical feedstocks (again only from 100% bio-based products at end-of-life) but ideally for production of high value materials and substances rather than cheaper energy products. Energy recovery is also not covered explicitly within the recirculation end-of-life definitions. Incineration is popular for energy recovery, and lessens the burden on landfill [39]. In some ways, incineration is more valuable than composting because of the favorable energy balance but composting has an environmental value [62]. What has happened in recent years is that energy recovery has become a major business, and this business thrives on the availability of low value waste. A mentality of this sort does not encourage a circular economy. To justify the practice of energy recovery, the type of application the product is used in should mean no other managed end-of-life option can be envisaged but to burn it (obviously, landfill will not be considered). To avoid the release of long cycle carbon to the atmosphere, contributing to increasing GHG emissions, only 100% bio-based products are suitable from the point of view of material recirculation.

**Figure 5:**
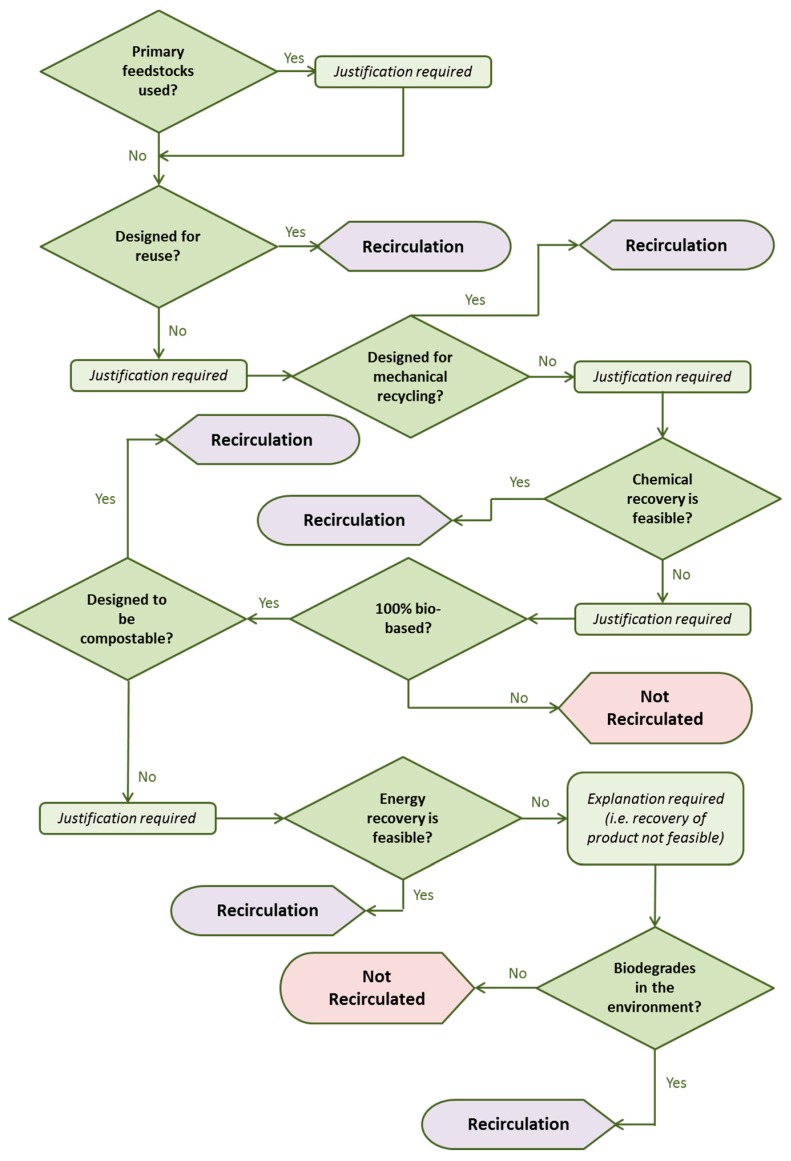
Decision flow chart for bio-based products designed to be recirculated (applies to each disassembled part).

Different options for recirculating products and their order of preference are clarified below (Figure 5). Reuse has a number of different forms. A design justification is required if the end-of-life option chosen is down the waste value hierarchy. Remanufacture can be considered as producing the same end result as closed loop recycling, but it is preferable because it is more direct and retains the form of the article or component part without having to reformulate or mold a recyclate back into its original form. Other types of reuse practice (i.e., extended lifespan through repair or reconditioning) are also strategies for maximum resource efficiency but do not constitute recirculation alone. A clear end-of-life plan is still required for the inevitable redundancy of the product when it occurs. Ultimately, a net loss in material resources indicates recirculation is not achieved. Recyclable products shall be designed so that they do not cause the depletion of the feedstocks needed for their manufacture simply because of design flaws.

### 3.3. Design Criteria

Standards would be one way to transform the recirculation philosophy into tangible instructions. This is then the basis for labelling criteria or certification, therefore translating definitions and ideas into a practical tool. A blueprint for such a standard has now been published on the Open-Bio project website [55]. Contained in the test method are design criteria, which are validated by pre-existing standards for different end-of-life practices as well as upstream issues. Essentially the design of a product shall maximize the possibility of (i) realizing the most efficient process of manufacturing (including the assembly of component parts) and (ii) the most appropriate waste management approach. To be more specific, the criteria to be met to demonstrate recirculation of a bio-based product covers three main areas:

- The design of the chosen manufacturing route, which should also reduce waste to a practical minimum;
- The product shall be designed to support the disassembly of any component parts in order to assist with the managed end-of-life options. When a product consists of multiple parts with different end-of-life pathways, disassembly must be achievable with the primary purpose of separating the component parts so that they can enter the correct waste streams without cross-contamination. Life cycle assessment (LCA) can be used as part of the justification, as can the functionality (e.g., food packaging limiting applications to a single use) in order to establish the most viable end-of-life process;
- The intended end-of-life pathway of a product or component, and its design for a maximum functioning lifetime, must not unnecessarily affect the performance of the entire article. The incorporation of bio-based content also creates new opportunities with respect to the performance of an article, with biomass and bio-based chemicals offering different functionality and new characteristics.

The recirculation design philosophy will require a major evaluation of many products. The design of a significant number of everyday articles has evolved through time to become high performance at the correct price for their market, but in doing so have become locked into a certain construction and assembly that cannot be easily reversed to prioritize keeping the material in use. Producers should expect, in a circular economy, to need to be in a position where the can justify product design choices should they come to restrict the use of secondary materials and biomass feedstocks, or encourage less preferable end-of-life options.

More often than not, multiple end-of-life treatments will be theoretically possible for any given product. It is the design of the product or component part in question that ultimately dictates which option is the most practical and preferable. In turn, the design is decided by the function and intended use of the product. The recirculation test method requires that the end-of-life practices that best retain the form and function of the product are prioritized, with a justification required each time a step is taken down the waste hierarchy (Figure 5) [63].

To illustrate the importance of design on recirculation, plastic lined disposable paper cups provide a contentious example. The article is made of two routinely recyclable materials, usually paper fiber adhered to an inner polyethylene film lining. The plastic lining solves the functionality problem of the water adsorbent paper, while the paper fiber forming the bulk of the mass provides the correct price, branding platform, rigidity, and fulfils consumer expectations regarding aesthetics etc. However, the article is not correctly designed for reuse, recycling, or biodegradation when the two materials are not easily and routinely separable for their specific recycling processes. It is only the design (barring economic factors) that is limiting recirculation in this example, where just a quarter of 1% of the three billion plastic lined disposable paper cups used in the UK every year are recycled [64]. A severe shortage of appropriate recycling centers is responsible [65,66].

To correct this problem, the use of a bio-based PLA film instead of polyethylene makes the entire article compostable [67], and so the product has now been designed in a way that allows for recirculation. One criticism is that reuse and recycling have been forsaken as target end-of-life options, and the recirculation test method encourages these higher value processes. A different design decision is to reduce the adhesive fastening and compensate with the addition of a releasable clasp-like system [68,69]. Consequently, this means the separation of the polyethylene plastic and paper components for recycling becomes routine. Although not a specific objective of the recirculation design criteria, should it not be possible to develop a product in order to improve its end-of-life waste management prospects then new recycling processes can be invented instead. Research has shown conventional polyethylene plastic lined disposable paper cups can be converted into a composite resin after use [70]. The paper element is actually beneficial to the performance of the composite, and so this is not just a way to ‘hide’ waste by burying it into plastics rather than in the ground.

With no disposable cup indisputably better for the environment outright than another [71], just discussing the variety of options might be helpful. It would provide flexibility to the waste management infrastructure, but we know from compostable plastics that the extra waste sorting required is a complex barrier to overcome. Furthermore, the sheer volume of plastic lined disposable paper cups exceeds the available recycling capacity and the market for secondary materials anyway [72]. Even if the required technology was more widely available the problem therefore would not be resolved. That is why we now have PLA containing biodegradable disposable cups.

What recirculation promotes above any other option in this case study is the rather obvious alternative of a reusable cup. At present, several coffee shop chains encourage customers to bring their own cup from home by offering a small discount [73]. Just a month of daily use puts the production energy balance in favor of a ceramic cup compared to using a plastic lined disposable paper cup each day [74]. To promote a cultural change towards cup reuse, an effort from all stakeholders in the value chain is needed, from producer, supplier, to consumer. The role of the recirculation concept in this is to clarify the benefit of design for different waste management options, indicating how the value inherent to materials can be best preserved beyond the lifespan of a single product.

## 4. Conclusions

The often repeated argument to use more renewable feedstocks and recycle greater quantities of waste is not in itself a feasible solution. Present day biorefineries produce products with a low biomass utilization efficiency [75], and the technical and economic feasibility of recycling and the size of the secondary materials market is very restrictive. These issues mean that material use is wasteful, prioritizing the function of primary products and often not attempting to provide valuable materials that last beyond a single use. The design of products, including the choice of feedstocks and what materials and substances they are converted into, has a hugely significant impact on the ability to keep that resource in use beyond the lifespan of the product.

The strengthening of a bio-based economy and exciting developments towards a circular economy mean the types of product we produce and how we make them is changing. We must take this opportunity to assess how we create functional molecules, materials, and final articles impacts the fate of that resource. An appreciation of product design is now starting to become evident with green chemists, with Paul Anastas [76], and David Constable [77], both writing recently on the topic. Our contribution is an elaboration to the waste hierarchy [63], with product design objectives to enable the most environmentally beneficial end-of-life process to be implemented [55].

## Figures and Tables

**Table 1:** European standards for bio-based products developed in CEN/TC 411 unless stated otherwise [8].

Standard	Title	Stage of Development ^a^
EN 16575	Bio-based products: Vocabulary	Published 2014.
CEN/TR 16721	Bio-based products: Overview of methods to determine the bio-based content	Published 2014.
CEN/TS 16640	Bio-based products: Determination of the bio based carbon content of products using the radiocarbon method	Published 2014.
CEN/TR 16957	Bio-based products: Guidelines for life cycle inventory (LCI) for the end-of-life phase	Published 2016.
FprEN 16640	Bio-based products: Bio-based carbon content—Determination of the bio-based carbon content using the radiocarbon method	Waiting approval.
EN 16785-1	Bio-based products: Bio-based content—Part 1: Determination of the bio-based content using the radiocarbon analysis and elemental analysis	Published 2015.
prEN 16785-2	Bio-based products: Bio-based content—Part 2: Determination of the bio-based content using the material balance method	Waiting approval.
EN 16760	Bio-based products: Life cycle assessment	Published 2015.
EN 16751	Bio-based products: Sustainability criteria	Published 2016.
EN 16848	Bio-based products: Requirements for business to business communication of characteristics using a data sheet	Published 2016.
FprEN 16935	Bio-based products: B2C reporting and communication—Requirements for claims	Waiting approval.
CEN/TS 16766	Bio-based solvents: Requirements and test methods	Published 2015.
EN 16807	Bio-lubricants: Criteria and requirements of bio-lubricants and bio-based lubricants	Published 2016. ^b^
CEN/TS 16398	Plastics: Template for reporting and communication of bio-based carbon content and recovery options of biopolymers and bioplastics—Data sheet	Published 2012. ^c^
FprCEN/TS 17035	Surface active agents: Bio-based surfactants—Requirements and test methods	Waiting approval. ^d^

^a^ As of October 201; ^b^ CEN/TC 19: fuels and lubricants; ^c^ CEN/TC 249: plastics; ^d^ CEN/TC 276: surface active agents.

**Table 2:** The 12 principles of green chemistry.

Principle	Meaning
Prevention	It is better to prevent waste than to treat or clean up waste after it has been created.
Atom economy	Synthetic methods should be designed to maximize the incorporation of all materials used in the process into the final product.
Less hazardous chemical synthesis	Wherever practicable, synthetic methods should be designed to use and generate substances that possess little or no toxicity to human health and the environment.
Designing safer chemicals	Chemical products should be designed to affect their desired function while minimizing their toxicity.
Safer solvents and auxiliaries	The use of auxiliary substances (e.g., solvents, separation agents, etc.) should be made unnecessary wherever possible and innocuous when used.
Design for energy efficiency	Energy requirements of chemical processes should be recognized for their environmental and economic impacts and should be minimized. If possible, synthetic methods should be conducted at ambient temperature and pressure.
Use of renewable feedstocks	A raw material or feedstock should be renewable rather than depleting whenever technically and economically practicable.
Reduce derivatives	Unnecessary derivatization (use of blocking groups, protection/deprotection, temporary modification of physical/chemical processes) should be minimized or avoided if possible, because such steps require additional reagents and can generate waste.
Catalysis	Catalytic reagents (as selective as possible) are superior to stoichiometric reagents.
Design for degradation	Chemical products should be designed so that, at the end of their function, they break down into innocuous degradation products and do not persist in the environment.
Real-time analysis for pollution prevention	Analytical methodologies need to be further developed to allow for real-time, in-process monitoring and control prior to the formation of hazardous substances.
Inherently safer chemistry for accident prevention	Substances and the form of a substance used in a chemical process should be chosen to minimize the potential for chemical accidents, including releases, explosions, and fires.

**Table 3:** Defining the recirculation of bio-based products.

Term	Definition
Recirculated	Returned to use within a certain timeframe by an anthropogenic process and/or a natural process.
Reusable	Returned to use within a certain timeframe without modification to the parent article or loss of performance.
Recyclable	Returned to use within a certain timeframe by an anthropogenic process.
Renewable	Comes from renewable resources and is returned to use within a certain timeframe by a natural process.
